# Inflammatory Cytokines, but Not Dietary Patterns, Are Related to Somatic Symptoms of Depression in a Sample of Women

**DOI:** 10.3389/fpsyt.2022.822466

**Published:** 2022-05-16

**Authors:** Danielle Belden Hazeltine, Ashley Rose Polokowski, Laura Christine Reigada

**Affiliations:** ^1^Department of Psychology, Brooklyn College, City University of New York, New York, NY, United States; ^2^Program in Psychology, The Graduate Center, City University of New York, New York, NY, United States; ^3^Psycho-Oncology, Monter Cancer Center, Northwell Health Cancer Institute, New York, NY, United States

**Keywords:** depression, inflammation, diet, cytokines, somatic, neurovegetative, nutrition

## Abstract

**Background:**

Depression is a heterogenous disorder with both cognitive and somatic symptom dimensions that may differentially relate to systemic inflammation. Diet, which has the potential to modulate both inflammation levels and mood, is yet to be studied within the context of individual depression dimensions. This study examined the associations between inflammatory cytokines and dietary patterns with depressive symptom dimension profiles among a sample of women recruited in a non-clinical setting.

**Methods:**

Inflammatory cytokines (IL-6 and TNF-α), inflammatory diet (Diet Inflammatory Index; DII), and depressive symptoms (Beck Depression Inventory-II; BDI-II) were measured in 136 females (*M*_*age*_ = 22.01 ± 4.02, range 18–59 years). Multiple linear regressions were used to investigate the relationships between inflammatory cytokines and diet with self-reported cognitive, somatic, and total depressive symptoms, adjusting for demographic factors.

**Results:**

Findings showed that increased somatic dimension scores were positively associated with IL-6 (ß = 0.273*, p* = 0.002) and TNF-α (ß = 0.215*, p* = 0.017), but not inflammatory diet (*p* = 0.300). Total BDI-II scores were only positively associated with IL-6 (ß = 0.221*, p* = 0.012), and cognitive dimension scores were not associated with any inflammation measures.

**Conclusions:**

These findings contribute to existing evidence that inflammatory cytokines are associated with the somatic symptoms of depression. Inflammatory diet index was not associated with depression measures.

## Introduction

Emerging evidence suggests that a subgroup of individuals with depression have elevated inflammation (1, 2). Inflammation, a biological process that fights infection, is brought on by groups of secreted proteins called cytokines (3, 4). Research has demonstrated positive correlations between cytokines interleukin (IL)-6 and tumor necrosis factor (TNF)-α and symptoms of depression in meta-analyses of both community samples (5, 6) and adults diagnosed with major depressive disorder (MDD) (2, 5, 7–11).

The evident relation between inflammation and depression is commonly explained by the sickness behavior model. Sickness behaviors are depression-like symptoms (fatigue, anhedonia, changes in sleep and appetite, and decreased interest in sex) resulting from infection or injury and subsequent rise in inflammation (12–15). These behaviors are evolutionary advantageous in that they promote sedentary behavior and eventual recovery from infection or injury. However, prolonged exposure to inflammation, and consequent sickness behaviors, may influence the development and maintenance of MDD (16).

In addition to infection or injury, a growing body of literature shows that an unhealthy diet may contribute to low-grade, prolonged systemic inflammation via pathways within the gut (17–19). An inflammatory diet has been associated with increased risk for depressive symptoms and diagnosis of MDD in a handful of studies (20–23), and thus, may be an important factor to consider when studying inflammation-associated depression (24). An unhealthy, inflammatory diet typically consists of highly processed foods such as white bread, sweets, sugary beverages, and large amounts of animal products high in saturated fats (25, 26). In contrast, a healthy, anti-inflammatory diet typically consisting of foods such as fruits, vegetables, whole grains, nuts, seeds, and fish, have been associated with low levels of depression (27–29). Based on these findings, diet may be one source of inflammation that contributes to sickness behaviors. Investigation of dietary intake that can contribute to inflammation is needed to better understand how lifestyle associated inflammation is associated with depression.

Historically, depression, as it relates to inflammation, has been examined as a global construct (30). In this way, prior research is limited, as depression encompasses a broad, heterogenous range of symptoms that may be differentially related to inflammation (31–34). Specifically, symptoms can be conceptualized as cognitive-affective (e.g., maladaptive beliefs or thinking patterns and sad mood) or somatic (e.g., fatigue, appetite, or sleep changes) (35). This distinction is relevant when considering the inflammation-depression link given that sickness behavior symptoms predominately overlap with somatic symptoms of depression, as opposed to cognitive symptoms of depression (33). Thus a dimensional analysis of depressive symptoms is a more nuanced approach to disentangle biological mechanisms of depression and their behavioral manifestations (31, 32). The somatic dimension has been shown to be associated with various inflammatory measures in both clinical (36, 37) and non-clinical samples (6, 34, 38, 39). However, not all studies demonstrate this association (40–44), and still others find equal associations between both dimensions and inflammation (6, 42). These inconsistent findings may be due to heterogeneity in methodology of how somatic symptoms are measured (45). Furthermore, depression research mostly measures inflammatory cytokines using blood samples. However, preliminary data shows that cytokine levels measured via saliva sampling, a non-invasive and more economical approach, (46) may be related to depression as well (47, 48).

A more multifaceted investigation, which includes measures of salivary cytokines, dietary patterns, and a dimensional assessment of depressive symptoms which has been validated by meta-analysis in previous research, is utilized in the current study to better elucidate the relationship between inflammation and depression. This study aimed to test the association between inflammatory cytokines and diet with depressive symptom dimensions (i.e., somatic and cognitive) in a community sample of women. It was predicted that inflammation measures would be associated with somatic symptoms of depression.

## Materials and Methods

### Participants and Procedure

We performed a secondary data analysis of baseline data collected from a randomized trial (RCT) testing the efficacy of a 12-week daily dose of supplements (i.e., omega-3 fatty acids, probiotics, combination, placebo) to reduced depressive, anxiety, and stress symptoms (49). This study was conducted at a college in Brooklyn, New York. Participants were recruited via flyers posted on campus, snowball sampling, and online advertisements. Inclusion criteria included self-reporting as a cisgender female, at least 18 years of age, and English fluency. Exclusion criteria included self-reported of any of the following: current pregnancy, a diagnosis of chronic inflammatory or endocrine related illnesses, a diagnosis of current schizophrenia or bipolar disorder, current psychoactive drug use, treatment (psychopharmacology or psychotherapy) for any psychiatric illness within 6 months prior to study recruitment, excessive alcohol or cigarette intake, more than occasional use of marijuana, or any use of other recreational drugs.

The research was approved by the university's institutional review board, and all participants provided informed, written consent prior to participation in research activities. In the lab, participants completed self-report assessments (demographic information, depressive symptoms, and diet), provided 0.5–2.0 ml of saliva via passive drool, and were compensated $25. Immediately after saliva collection, samples were placed in a −20 degrees Celsius freezer for approximately 3–6 months. They were then mailed to Salimetrics Laboratory on dry ice for analysis (Salimetrics, LLC, State College, Pennsylvania).

### Measures

#### Demographic Variables

Participants were asked to report their age, race/ethnicity, and income. Age was measured on a continuous scale. For race/ethnicity, participants had the option of choosing Caucasian, Hispanic/Latina, Black/African American, Asian/Pacific Islander, or Mixed/Other. Income was assessed using seven income brackets ranging from < $25,000 to $150,000 or more.

#### Beck Depression Inventory (BDI-II)

The BDI-II assesses severity of depressive symptoms using a 21-items questionnaire (45). Total scores can range from 0 to 63, with higher scores indicative of more severe depression symptoms. Established clinical cutoff scores include normal ranges (0–13), mild depression (14–19), moderate depression (20–28), and severe depression (29 or above). The BDI-II has adequate validity (50). In the current sample, total scores had good internal consistency (Cronbach's α = 0.89).

To calculate somatic and cognitive dimensions of depression, Huang & Chen's (51) method was used. Briefly, the authors conducted a meta-analysis of the factor structure of the BDI-II's depression dimensions which resulted in the following two-factor structure. The somatic dimension is a summed score of 11 items on the BDI-II (items 4, 11–13, and 15–21). The cognitive dimension is a summed score of nine items on the BDI-II (items 1–3, 5–9, and 14). Scores obtained in the current study suggest that the somatic and cognitive dimensions have good internal consistency (Cronbach's α = 0.81 and 0.86, respectively).

#### Salivary Cytokines

Prior to initiating in-person data collection, participants were instructed to refrain from drinking alcohol and smoking 12 h prior to their study appointment, and to refrain from eating, consuming caffeine, and brushing their teeth within 1 h of their appointment to ensure more accurate measurement of salivary inflammatory cytokines. Instruction compliance was verified before initiating any study procedures. Salivary inflammation was measured via passive drool.

A total of 136 saliva samples were analyzed by Salimetrics Laboratory (Salimetrics, LLC, State College, Pennsylvania). Samples were centrifuged at 3,500 rpm for 15 min, and supernatant was transferred away from the resultant mucin and debris. Next, supernatant samples were mixed again by inversion and vortexing and then stored in a laboratory freezer set at −80 degrees Celsius until they were assayed. Enzyme-linked immunosorbent assay (ELISA) was used to detect each participant's average levels of IL-6 and TNF-α in pg/mL. Minimum detection limits for IL-6 were 0.07 pg/mL. IL-6 values in the sample ranged from 0.07 to 92 pg/mL, mean (SD) = 5.79 (11.52) pg/mL, which is comparable to other, larger samples (36, 42, 52). Minimum detection limits for TNF-α were 0.04 pg/mL. TNF-α values in the sample ranged from 0.22 to 336.98 pg/mL, mean (SD) = 10.42 (43.53) pg/mL, which is comparable to other, larger samples (52, 53). ELISA procedures performed by Salimetrics were reported to be reliable via several parameters including linearity, spike and recovery testing, intra- and inter- assay precision, and freeze/thaw stability (54).

#### Inflammatory Diet

Measurement of the inflammatory potential of participants' diets was based on self-reported dietary intake using the Diet History Questionnaire-II [DHQ-II; (55)]. The DHQ-II (56) is a web-based, self-reported food frequency questionnaire that assesses intake of 134 food and beverage items and eight dietary supplements over the past month in terms of both frequency and portion size. DHQ-II items were transformed using the U.S. Department of Agriculture's (USDA) Food and Nutrient Database for Dietary Studies and USDA's MyPyramid Equivalents Database (MPED) to produce an average daily amount for food items and nutrients. This method is commonly used in nutrition research that uses the DHQ-II (57). A software program, Diet^*^Calc PC [Version 1.5, (58)] was utilized to convert data generated by the DHQ-II into an estimated value of daily nutrient consumption in grams (59, 60).

**The Diet Inflammatory Index (DII)** was used to determine the inflammatory potential of participants' diets (61). Briefly, the DII is a validated, literature-derived index used to calculate the inflammatory potential of participants' self-reported dietary patterns (62, 63). The methods outlined by Shivappa et al. (61) to calculate the total DII scores were used. A total of 27 food and nutrient items obtained from the DHQ-II were used in this study, including alcohol, beta-carotene, caffeine, carbohydrates, cholesterol, total fat, fiber, folic acid, iron, magnesium, monounsaturated fatty acids, niacin, omega-3, protein, polyunsaturated fatty acids, riboflavin, saturated fats, selenium, thiamin, trans fats, vitamins A, B12, B6, C, D, E, and zinc. These items were adjusted for total energy intake using the residual method (64), and then summed. DII scores closer to −5 indicate a more anti-inflammatory diet, and scores closer to +5 indicate a more pro-inflammatory diet.

### Data Analysis

Statistical analysis was conducted using Statistical Package for the Social Sciences (SPSS) version 27 (65). First, data was examined for normality and outliers. While depression variables were normally distributed, distributions of IL-6 and TNF-α values were significantly skewed (Skapiro-Wilk = 0.40 and 0.55, respectively), these scores were transformed using a natural logarithmic transformation to normalize the data. Two outliers were identified for IL-6 (92 and 60 pg/mL), and 1 for TNF-α (44 pg/mL). Outliers were windsorized by replacing values at or above 3 standard deviations above the mean with the next highest value (66). These transformation methods for cytokines are commonly used within the literature (52, 67). Alpha was set at 0.05 for all analyses.

Two-tailed Pearson correlations tested preliminary associations between the three predictor variables (DII, IL-6, and TNF-α) and the depression dimensions and total BDI-II scores. The univariate relationship between depression and inflammation was studied by hierarchical linear regression analyses. The somatic dimension, cognitive dimension, and total BDI-II scores were examined as separate dependent variables. First, inflammation level (IL-6, TNF-α) and DII score were entered as the independent variables separately, and then entered together. For models with independent variables entered together, multicollinearity was ruled out by calculating variance inflation factors (VIF). VIFs for IL-6, TNF-α, and DII were 1.96, 2.00, and 1.06, respectively.

Regression models were adjusted for demographic factors (age, income, and race/ethnicity). For all regression analyses, demographic factors were entered into the first step and inflammation measures were entered in the second step. Income was represented as a scale of 1–7, with each point representing one of the seven income groups. Racial/ethnic groups were represented as dummy variables with White as the reference group.

## Results

The sample included 136 adult female participants with ages ranging from 18 to 59 years old (*M* = 22.24, *SD* = 4.96). Table 1 shows a description of participants' demographic characteristics, and the descriptive statistics for depression, inflammation, and diet measures. Participants were racially/ethnically and economically diverse, with more than three-quarters of the participants identifying as Latina (23.5%), Black (19.9%), or Asian/Pacific Islander (19.1%) and a wide range of annual household income levels. Total BDI-II scores ranged from 0 to 51, the somatic dimension ranged from 0 to 26, and the cognitive dimension ranged from 0 to 22. Although this sample was recruited in a non-clinical setting, 40.4% (*N* = 55) of participants scored within the clinically significant range of symptom severity in the BDI-II. More specifically, based on clinical cutoff scores on the BDI-II, 29 (21.3%) participants reported mild clinical depressive symptoms, 20 (14.7%) reported moderate clinical depressive symptoms, and 6 (4.4%) scored in the severe clinical depressive symptom range. Participants had a mean DII score of 0.0 (*SD* = 2.02) with a range of −5 to +4.48, indicating that the average diet reported was neither pro- or anti-inflammatory.

**Table 1:** Participants' demographic characteristics (*N* = 136).

**Characteristics**	**Mean ±SD or *N* (%)**
Age (in years)	22.01 ± 4.02
Sex	
Female	136 (100.0)
Race/ethnicity	
Caucasian	31 (22.8)
Hispanic/Latina	32 (23.5)
Black/African American	27 (19.9)
Asian/Pacific Islander	26 (19.1)
Other/mixed	20 (14.7)
Annual income	
< $25,000	47 (34.6)
$25,000–$34,999	17 (12.5)
$35,000–$49,999	19 (14.0)
$50,000–$74,999	24 (17.6)
$75,000–$99,999	10 (7.4)
$100,000–$149,999	9 (6.6)
$150,000 or more	9 (6.6)
Depression symptoms	
Total BDI-II	12.54 ± 8.97
Somatic dimension	7.74 ± 5.11
Cognitive Dimension	4.39 ± 4.42
Inflammation measures	
IL-6	5.79 ± 11.52; range: 0–92
Transformed IL-6*	1.10 ± 0.93
TNF-α	4.08 ± 5.93; range: 0.22–44.32
Transformed TNF-α^*^	0.88 ± 0.95
DII	0.00 ± 2.02; range:−5.00–+4.48

*BDI-II (Beck Depression Inventory II), IL-6 (Interleukin-6), TNF-α (Tumor Necrosis Factor-α), DII (Diet Inflammatory Index)*.

*Log transformed and windsorized.

Table 2 shows bivariate correlations between depression and inflammation variables. Depression variables were positively correlated with each other, and inflammatory cytokines were positively correlated with each other. Inflammatory cytokines IL-6 and TNF-α were positively correlated with the somatic dimension, but not cognitive. IL-6 was positively correlated with total BDI-II scores. DII scores were not correlated with any measure.

**Table 2:** Bivariate correlations of depression, cytokines, and diet.

	**Total BDI-II**	**Somatic dimension**	**Cognitive dimension**	**IL-6**	**TNF-α**
Somatic dimension	**0.91 (<0.001)**				
Cognitive dimension	**0.89 (<0.001)**	**0.63 (<0.001)**			
IL-6	**0.19 (0.03)**	**0.25 (0.003)**	0.08 (0.39)		
TNF-α	0.13 (0.14)	**0.20 (0.02)**	0.05 (0.56)	**0.70 (<0.001)**	
DII	0.06 (0.46)	0.09 (0.30)	−0.01 (0.95)	0.07 (0.40)	0.05 (0.60)

*Bolded correlations p < 0.05*.

*BDI-II (Beck Depression Inventory II), IL-6 (Interleukin-6), TNF-α (Tumor Necrosis Factor-α), DII (Diet Inflammatory Index)*.

### Associations Between Inflammation and Depression

Results of demographic-adjusted models for the association between salivary cytokines and DII scores entered separately, and depression variables are presented in Table 3. Multiple regression results show that higher IL-6 was associated with report of more somatic symptoms of depression (ß = 0.273*, p* = 0.002) and higher total BDI-II scores (ß = 0.221*, p* = 0.012). IL-6 was not associated with cognitive symptoms of depression. Likewise, increased TNF-α was positively associated with the presence of more somatic symptoms (ß = 0.215*, p* = 0.017). TNF-α was not associated with the cognitive dimension nor total BDI-II scores. No significant association was observed between the DII and depression measures.

**Table 3:** Summary of multiple regression analysis examining the adjusted relationship between inflammation measures and dimensions of depression and total BDI-II scores.

	**Somatic dimension**		**Cognitive dimension**		**Total BDI-II**	
**Inflammation**	**ß**	***p***	**ß**	***p***	**ß**	***p***
**Measure**						
IL-6	**0.273**	**0.002**	0.120	0.168	**0.221**	**0.012**
TNF-α	**0.215**	**0.017**	0.062	0.485	0.145	0.108
DII	0.083	0.359	−0.023	0.795	0.048	0.591

*ß: standardized coefficients*.

*Significant associations are highlighted in bold font. All tests were two-tailed. Models were adjusted for age, income, and race/ethnicity. Income was represented as a scale of 1–7, with each point representing one of the seven income groups. Race/ethnicity was represented as four dummy variables, with White as the reference group*.

*Bolded associations p < 0.05*.

*IL-6 (Interleukin-6), TNF-α (Tumor Necrosis Factor-α), DII (Diet Inflammatory Index)*.

Tables 4–6 show the results of demographic-adjusted models for the association between salivary cytokines and DII scores entered together, and depression variables. Overall, no model examining inflammation variables together were associated with depression variables. No covariates were significantly associated with depression variables in these models, although IL-6 approached significance (ß = 0.235, *p* = 0.052).

**Table 4:** Summary of multiple regression analysis examining the adjusted relationship between inflammation variables and the somatic dimension.

	**Unstandardized coefficients**		**Standardized coefficients**					
**Predictor**	***B***	**SE**	**ß**	***p***	***R* ^2^**	***ΔR^**2**^***	***F***	***p***
**Model 1:**					0.008	0.008	0.168	0.985
Age	−0.108	0.121	−0.085	0.376				
Income	−0.037	0.239	−0.014	0.879				
Hispanic/Latina	−0.308	1.334	−0.026	0.818				
Black/African American	−0.289	1.404	−0.023	0.837				
Asian/Pacific Islander	−0.583	1.403	−0.045	0.678				
Other/mixed	−0.004	1.625	−0.000	0.998				
**Model 2:**					0.085	0.077	1.282	0.253
Age	−0.125	0.120	−0.098	0.299				
Income	0.012	0.237	0.005	0.958				
Hispanic/Latina	−0.263	1.300	−0.022	0.840				
Black/African American	−0.000	1.403	0.000	1.000				
Asian/Pacific Islander	−0.145	1.376	−0.011	0.916				
Other/mixed	−0.173	1.592	−0.012	0.914				
IL-6	1.333	0.679	0.235	0.052				
TNF–α	0.272	0.668	0.049	0.685				
DII	0.152	0.223	0.060	0.498				

*SE: standard error of B*.

*ß: standardized coefficients*.

*ΔR^2^: change in R^2^*.

*Models were adjusted for age, income, and race/ethnicity. Income was represented as a scale of 1–7, with each point representing one of the seven income groups. Race/ethnicity was represented as four dummy variables, with White as the reference group*.

*Bolded associations p < 0.05*.

*IL-6 (Interleukin-6), TNF-α (Tumor Necrosis Factor-α), DII (Diet Inflammatory Index)*.

**Table 5:** Summary of multiple regression analysis examining the adjusted relationship between inflammation variables and the cognitive dimension.

	**Unstandardized coefficients**		**Standardized coefficients**					
**Predictor**	***B***	**SE**	**ß**	***p***	***R* ^2^**	***ΔR^**2**^***	***F***	***p***
**Model 1:**					0.060	0.060	1.357	0.237
Age	−0.129	0.102	−0.117	0.210				
Income	0.292	0.201	0.127	0.149				
Hispanic/Latina	−0.177	1.125	−0.017	0.875				
Black/African American	−1.354	1.184	−0.122	0.255				
Asian/Pacific Islander	0.165	1.183	0.015	0.889				
Other/mixed	−1.039	1.370	−0.083	0.450				
**Model 2:**					0.076	0.016	1.137	0.342
Age	−0.143	0.104	−0.130	0.172				
Income	0.342	0.207	0.148	0.100				
Hispanic/Latina	−0.187	1.131	−0.018	0.869				
Black/African American	−1.318	1.220	−0.119	0.282				
Asian/Pacific Islander	0.261	1.197	0.023	0.828				
Other/mixed	−1.040	1.385	−0.084	0.454				
IL-6	0.740	0.591	0.151	0.213				
TNF–α	−0.193	0.581	−0.040	0.740				
DII	−0.075	0.194	−0.034	0.702				

*SE: standard error of B*.

*ß: standardized coefficients*.

*ΔR^2^: change in R^2^*.

*Models were adjusted for age, income, and race/ethnicity. Income was represented as a scale of 1–7, with each point representing one of the seven income groups. Race/ethnicity was represented as four dummy variables, with White as the reference group*.

*Bolded associations p < 0.05*.

*IL−6 (Interleukin−6), TNF–α (Tumor Necrosis Factor–α), DII (Diet Inflammatory Index)*.

**Table 6:** Summary of multiple regression analysis examining the adjusted relationship between inflammation variables and total BDI-II scores.

	**Unstandardized coefficients**		**Standardized coefficients**					
**Predictor**	***B***	**SE**	**ß**	***p***	***R* ^2^**	***ΔR^**2**^***	***F***	***p***
**Model 1:**					0.027	0.027	0.581	0.745
Age	−0.272	0.211	−0.122	0.201				
Income	0.323	0.416	0.069	0.439				
Hispanic/Latina	−0.269	2.321	−0.013	0.908				
Black/African American	−1.451	2.443	−0.065	0.554				
Asian/Pacific Islander	−0.304	2.441	−0.013	0.901				
Other/mixed	−0.853	2.828	−0.034	0.763				
**Model 2:**					0.074	0.048	1.117	0.356
Age	−0.298	0.212	−0.133	0.163				
Income	0.424	0.419	0.090	0.314				
Hispanic/Latina	−0.268	2.295	−0.013	0.907				
Black/African American	−1.243	2.477	−0.055	0.617				
Asian/Pacific Islander	0.204	2.429	0.009	0.933				
Other/mixed	−1.099	2.812	−0.044	0.697				
IL-6	2.277	1.199	0.229	0.060				
TNF-α	−0.149	1.179	−0.015	0.899				
DII	0.130	0.394	0.029	0.743				

*SE: standard error of B*.

*ß: standardized coefficients*.

*ΔR^2^: change in R^2^*.

*Models were adjusted for age, income, and race/ethnicity. Income was represented as a scale of 1-7, with each point representing one of the seven income groups. Race/ethnicity was represented as four dummy variables, with White as the reference group*.

*Bolded associations p < 0.05*.

*BDI-II (Beck Depression Inventory-II), IL-6 (Interleukin-6), TNF-α (Tumor Necrosis Factor-α), DII (Diet Inflammatory Index)*.

## Discussion

This study investigated the relationships between inflammatory cytokines and diet with dimensions of depressive symptoms among a diverse (i.e., socio-economic, race, and ethnicity) sample of women recruited in a non-clinical setting. Salivary inflammatory markers were found to be positively associated with the somatic dimension of depression when examined as separate independent variables. This finding parallels the results of other studies utilizing majority-White samples that found increased somatic symptoms, but not cognitive symptoms, to be associated with blood levels of IL-6 (39), TNF-α (36), and other inflammatory cytokines (6, 40, 68–71). These findings are also in concordance with the sickness behavior model, which posits that somatic depressive symptoms (sickness behavior) are related to immune system activity (12–15). After accounting for overlapping contributions, no inflammatory marker remained significant.

This study is the first to examine the relationship between dimensions of depressive symptoms and a measure of dietary patterns. Varying levels of an inflammatory diet pattern were not associated with depressive symptoms nor salivary cytokines. These findings contradict a review of six studies which found the DII to be positively associated with depressive symptoms in women (23), as well as existing research showing a relationship between the DII and biological markers for inflammation (62, 63, 72, 73). However, the present sample is racially and ethnically diverse, which has limited representation in the previous research, and signal the need for further attention. For instance, existing self-report measures of diet may not accurately or equally assess food consumption across different races or ethnicities (74–79) because diet composition can vary by culture (80); thus, future research with racially and ethnically diverse samples should include diet assessments that can capture culturally specific foods that have inflammatory potential. Furthermore, this study is of the first to examine salivary inflammation markers in relation to the DII. Future research comparing blood and salivary measures of inflammatory markers is needed. Of note, diet quality, inflammatory biomarkers, and depression are also associated with oxidative stress (81), absorption of specific vitamins (82, 83), psychological stress (84), gut microbiome health (85), and preferences for unhealthy foods (86). Collectively, this suggests that the relationships between the inflammatory potential of a diet, the actual level of inflammatory biomarkers observed, and depression is complex.

The current study is one of few that has examined the relationships between multiple inflammatory measures and meta-analysis derived depression dimensions of the BDI-II (87–89). There is a large amount of heterogeneity across research methods measuring somatic symptoms of depression within studies, making it challenging to draw distinct conclusions (51). For example, when using the BDI-II, some researchers rely on dimensions established in early dimension analyses of the BDI-II (38, 40) and others use dimensions that arise from data reduction techniques within their individual study (90, 91). Operationalizing dimensions consistently across studies is necessary to draw valid conclusions about the relationships between inflammatory markers and the somatic dimension of depression.

As this sample included only females, sex was controlled for. Prior research found females to be more susceptible to elevated inflammation (92), major depressive disorder (93), as well as inflammation-associated depressive symptoms (92, 94) which points to the importance of controlling for sex when studying the relationship between diet, inflammation, and depression. One limitation to an all-female sample, however, is that results may not be generalizable to males. This study was conducted in a non-clinical setting and examined the full range of depressive symptoms. However, 40% of participants reported scores above the clinical cutoff on the BDI-II, suggesting that they possibly meet criteria for a mood disorder. Community rates of individuals scoring above the clinical cutoff on the BDI-II ranged from 26% in racially/ethnically diverse student samples (95) to 30% in samples of Black adults (96). Rates may be higher in this current sample due to the intersectionality between female gender and racial/ethnic identity, as both are associated with greater vulnerability to depressive symptoms related to socio-cultural factors (97).

Several additional limitations should be considered. First, this study was cross-sectional precluding conclusions or interpretations about causality. This study did not account for time of day nor acute infection related to oral health, or otherwise, when collecting saliva samples. Salivary cytokine concentrations have been shown to have a diurnal rhythm (98) and be influenced by acute infection in the mouth such as gingivitis (99). There are known biases in self-report measures of dietary consumption including recall errors and social desirability factors (100, 101). Another limitation of this study is that it did not assess tobacco use, alcohol intake, or body mass index (BMI) as covariates.

In conclusion, this study provides support that both IL-6 and TNF-α are related to a meta-analysis derived somatic dimension of depression. A self-reported measure for an inflammatory diet pattern may be a less robust predictor of “inflamed depression” than salivary cytokines. Research aimed at phenotyping inflamed-depression subtypes should consider using consistent dimensions of depression, testing these relationships longitudinally, and examining additional biological factors such as gut microbiome health and nutrient absorption.

## Data Availability Statement

The raw data supporting the conclusions of this article will be made available by the authors, without undue reservation.

## Ethics Statement

The studies involving human participants were reviewed and approved by Brooklyn College Institution Review Board Human Research Protection Program. The patients/participants provided their written informed consent to participate in this study.

## Author Contributions

DH contributed to conceptualization, writing (original draft, reviewing, and editing), formal analysis, and data curation. AP contributed to methodology, writing (reviewing and editing), investigation, and funding acquisition. LR contributed to conceptualization, methodology, writing (reviewing and editing), and supervision. All authors contributed to the article and approved the submitted version.

## Funding

This work was supported by the Heppelmann Wacek Charitable Gift Fund.

## Conflict of Interest

The authors declare that the research was conducted in the absence of any commercial or financial relationships that could be construed as a potential conflict of interest.

## Publisher's Note

All claims expressed in this article are solely those of the authors and do not necessarily represent those of their affiliated organizations, or those of the publisher, the editors and the reviewers. Any product that may be evaluated in this article, or claim that may be made by its manufacturer, is not guaranteed or endorsed by the publisher.
